# Thermal Effect during Laser-Induced Plasmonic Heating of Polyelectrolyte-Coated Gold Nanorods in Well Plates

**DOI:** 10.3390/nano13050845

**Published:** 2023-02-24

**Authors:** Sujin Jiracheewanun, Michael B. Cortie, Dakrong Pissuwan

**Affiliations:** 1 Department of Mechanical Technology Education, Faculty of Industrial Education and Technology, King Mongkut’s University of Technology Thonburi, Bangkok 10400, Thailand; 2 School of Mechanical, Materials, Mechatronics and Biomedical Engineering, University of Wollongong, Wollongong 2522, Australia; 3 Materials Science and Engineering Program, Faculty of Science, Mahidol University, Bangkok 10400, Thailand; 4 Nanobiotechnology and Nanobiomaterials Research (N-BMR) Laboratory, School of Materials Science and Innovation, Faculty of Science, Mahidol University, Bangkok 10400, Thailand

**Keywords:** gold nanorods, optical properties, photoconductivity, polyelectrolyte

## Abstract

We examined the generation and transfer of heat when laser irradiation is applied to water containing a suspension of gold nanorods coated with different polyelectrolytes. The ubiquitous well plate was used as the geometry for these studies. The predictions of a finite element model were compared to experimental measurements. It is found that relatively high fluences must be applied in order to generate biologically relevant changes in temperature. This is due to the significant lateral heat transfer from the sides of the well, which strongly limits the temperature that can be achieved. A 650 mW continuous-wave (CW) laser, with a wavelength that is similar to the longitudinal plasmon resonance peak of the gold nanorods, can deliver heat with an overall efficiency of up to 3%. This is double the efficiency achievable without the nanorods. An increase in temperature of up to 15 °C can be achieved, which is suitable for the induction of cell death by hyperthermia. The nature of the polymer coating on the surface of the gold nanorods is found to have a small effect.

## 1. Introduction

Gold nanorods (GNRs) have recently attracted attention in diagnostic and therapeutic applications due to their unique optical properties [1,2,3]. Specifically, they exhibit two peaks in their optical absorption spectra, one at about 520 to 530 nm due to transverse plasmon resonance (SPR_T_) and a second strong peak that can be tuned to the near-infrared by controlling the nanorod shape. The latter is due to longitudinal plasmon resonance (SPR_L_) and has a higher extinction coefficient than that of the transverse band because its electronic oscillation is aligned through the length of the GNRs. Owing to these properties, GNRs exhibit higher light absorption than spherically shaped gold nanoparticles or gold nanoshells [4]. Furthermore, the longitudinal band of GNRs can be tuned to match the desired peak absorption and application. To synthesize GNRs, the toxic cationic surfactant cetyltrimethylammonium bromide (CTAB) is generally used. Therefore, the modification of the surface of as-synthesized GNRs is needed for the preparation of biocompatible GNRs. Additionally, the materials used for coating the surface of GNRs should help maintain the GNRs’ colloidal stability. Many approaches have been used to modify the surface of GNRs. One technique that is widely used is coating with polyelectrolytes or polymers [5,6]. Recently, various biocompatible polyelectrolyte-coated GNRs have been used in photothermal therapeutic applications [7,8,9,10,11]. There have been a number of experiments and modeling reports on the photothermal conversion efficiency of GNRs exposed to a laser in vitro, for example, Ref. [12].

There are two very different mechanisms by which GNRs can enhance laser-induced heating in a therapeutic context. In the first, the GNRs are irradiated with such a high intensity of laser light that they melt or even explode [13]. In this case, the target cells (whether a pathogen or cancer) are destroyed by physical damage such as perforation. This mechanism is sometimes referred to as thermal ablation and requires that the GNRs be located very close to the target cells. An alternative strategy is to use the suspension of GNRs to provide an overall increase in the optical absorptance of the target environment. Close contact between the GNRs and the target cells is neither required nor directly useful in this case; instead, the destructive payload is delivered by a diffuse localized increase in temperature. It can be sufficient to increase the temperature by only a few tens of Celsius, at which point the target cells or pathogens are killed by heat stress. This is the strategy that we explore in the present paper.

Information on the photothermal conversion efficiency and environmental effects resulting from the heating of GNRs is useful in designing GNRs suitable for photothermal applications. Surprisingly, although in vitro photothermal experiments are very often performed in 96-well plates, it appears that a specific examination of the heat transfer for the well-plate geometry is still lacking. In addition, much more information is available on the use of nanoparticles for thermoablation than for hyperthermia. Better information on the photothermal conversion efficiency and heat flow resulting from the heating of GNRs in the hyperthermal scenario applied to well plates could guide future experimental protocols and studies of therapeutic efficacy. This is the problem that we address in this study.

GNRs coated with poly(sodium 4-styrenesulfonate) (PSS), poly(diallyldimethylammonium chloride) (PDAC), and poly(allylamine hydrochloride) (PAH) were designed as models for the photothermal conversion study. These three polyelectrolytes were used because they have been commonly applied in photothermal therapy [14,15,16]. The results of the measurements were then used to inform multiphysics finite element calculations. This analysis identifies the absorption coefficients (α) and provides insight into the heating process. The heat transfer conduction equation used in the thermal modeling, which included water and the GNR ensemble, will be presented later. Briefly, the heat source is taken to be the energy of the laser light that is absorbed in the thickness of the liquid, minus the outflow of heat into the structures surrounding the hole, following the law of energy conservation [12,17]. The investigation was performed in a 96-well plate because it is commonly used as a vessel for in vitro photothermal studies. The wavelength used in the investigations (635 nm) was selected because it is just inside the so-called tissue window [18] and close enough to the SPR_L_ of the polyelectrolyte-coated GNRs used in this work. The results of this work could be useful in developing polyelectrolyte-coated GNRs for photothermal applications.

## 2. Experimental Setup and Numerical Analysis

### 2.1. Preparation of Polyelectrolyte-Coated GNRs

GNRs with the original surface coating of the cationic detergent cetyltrimethylammonium bromide, ~25 nm in width and ~47 nm in length, from Nanopartz (Nanopartz Inc., Loveland, CO, USA) were modified with PSS, PAH, and PDAC (Sigma-Aldrich Pte. Ltd., Ascent, Singapore) by slightly altering and refining the approach described in [5,6]. This modification occurs by means of an electrostatic attraction. Briefly, 600 µL of GNRs was mixed with 300 µL of PSS. The mixture was shaken for 30 min and then centrifuged. Finally, GNRs modified with anionic PSS (PSS-GNRs) were obtained. To prepare the GNRs modified with PAH (PAH-GNRs), a solution of 500 µL of PSS-GNRs was mixed with 15 µL of 10 mg mL^−1^ PAH (dissolved in 10 mM NaCl) for 30 min. Subsequently, the mixture was centrifuged, and the PAH-GNRs were finally obtained. GNRs modified with PDAC (PDAC-GNRs) were prepared using an approach similar to that used for PAH-GNRs. However, 20 µL of 2 mg mL^−1^ PDAC (dissolved in 1 mM NaCl) was added to the PSS-GNRs. The characterization of GNRs was carried out using UV-Vis spectrophotometry (Shimadzu, Nagagyo-ku, Kyoto, Japan) and a zeta potential analyzer (Malvern Panalytical Ltd., Malvern, United Kingdom).

### 2.2. Irradiation

**Figure 1:**
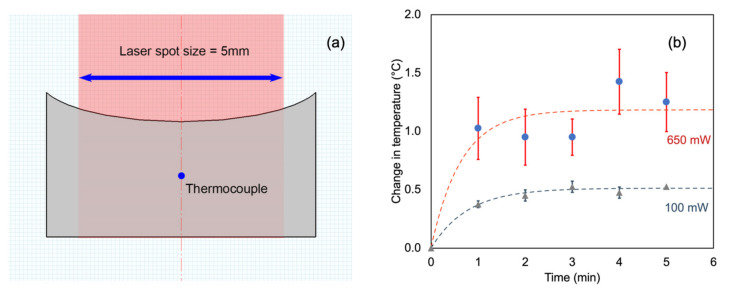
(**a**) Schematic representation of the position of the thermocouple. (**b**) The increase in temperature (from room temperature) measured when exposing the thermocouple to the laser in air. An exponentially decaying heating model has been fitted in each case to guide the eye (n = 4).

The photothermal conversion study of the three different colloids of surface-modified GNRs was performed by pipetting 30 or 100 μL of the GNR solution into a well of a 96-well plate. The suspension of GNRs used in this work was adjusted to have an OD value of 1.0 at OD_605nm_. This corresponds to a concentration of ~5.2 × 10^11^ GNR nanoparticles mL^−1^ for the particular particles used. (The calibration of particle density vs. OD is supplied by the vendor of the GNRs.) Following this, the GNR solution was irradiated with a continuous-wave (CW) laser (wavelength 635 ± 5 nm; CNI Laser, http://www.cnilaser.com (accessed on 4 December 2022), Changchun, China). The size of the laser spot is ~5 mm. The laser wavelength used overlapped sufficiently with the longitudinal plasmon resonance of the GNRs used (discussed later). The laser power applied was ~100 mW (for the 30 µL GNR solution) and 650 mW (for the 100 µL GNR solution), and the exposure time was 5 min. This exposure was achieved by setting the position of the laser on the top of each well. To prevent any impacts of light scattering or heat conduction to or from nearby wells, the test wells were well separated from one another. The temperature was measured using a digital thermocouple Fluke 54 II B (Fluke Corporation, Everett, WA, USA) and the diameter of the thermocouple is ~0.5 mm) by positioning the thermocouple halfway between the top and bottom of the liquid domain. The geometry of the well and laser irradiation is shown in Figure 1a. The diameter of the well is 6.5 mm. The heights of the liquid were ~0.85 mm (for 30 μL) and ~2.8 mm (for 100 μL). Under these conditions, the relevant therapeutic mechanism would be hyperthermia (low or moderate temperature) rather than thermoablation (high temperature; T > 55 °C) [19,20,21].

The absorption and scattering of the laser beam by the thermocouple are potential complicating factors. The absorption of energy by the thermocouple was tested first in the air at 100 and 650 mW (Figure 1b). Since there was only about a 0.5 °C increase in the temperature of the thermocouple in the 100 mW case, the thermocouple was left in place for the 30 μL experiments. The net ΔT was obtained by subtracting the temperature rise obtained for a control consisting of a well containing only pure water and the thermocouple. In the case of the 100 μL experiments (which were carried out at 650 mW), irradiation was applied for 5 min without the thermocouple present, and the thermocouple was introduced the instant the laser was switched off.

### 2.3. Calculation

The average photothermal conversion efficiency (PTE, η) is defined as the ratio of the increase in the internal energy of the fluid to the total radiation input (as shown in Equation (1)):

(1) $$\eta =\frac{\left(m_{\mathrm{Au}}\cdot c_{p,\mathrm{Au}}+m_{w}\cdot c_{p,w}\right)\Delta T\;}{Q_{\mathrm{laser}}}\approx \frac{\left(m_{w}\cdot c_{p,w}\right)\Delta T\;}{Q_{\mathrm{laser}}}$$

where $m_{\mathrm{Au}}{,\;c}_{p,\mathrm{Au}}{,\;m}_{w}{,\;c}_{p,w},\;\mathrm{and}\;\Delta T$ are the mass of gold nanorods, the heat capacity of gold nanorods, the mass of water, the heat capacity of water, and the average change in temperature after irradiation, respectively. The total heat source, $Q_{\mathrm{laser}}$, is calculated by multiplying the laser power and irradiation time. The thermal energy partitioned to the gold nanoparticles (m_Au_·c_p.Au_ΔT) is negligible because of their extremely low concentration, reaching only 4 × 10^−5^ J/K in 1 mL of suspension compared to 4.182 J/K for 1 mL of the surrounding water. It can therefore be neglected. The net heat absorbed by GNRs during laser irradiation was calculated using the optical absorption coefficient of the GNR/polyelectrolyte particles, with the assumption that absorbed light is converted to heat. The heat generation and heat transfer were simulated using the commercial package COMSOL Multiphysics (version 5.1, COMSOL Multiphysics, Stockholm, Sweden) to determine the effect of the laser power intensity. The software uses the finite element method to solve partial differential equations. In the liquid domain, convection was included as buoyancy-driven flow in the momentum equation (*z*-direction).

## 3. Results and Discussion

### 3.1. Polyelectrolyte-Coated GNRs

**Figure 2:**
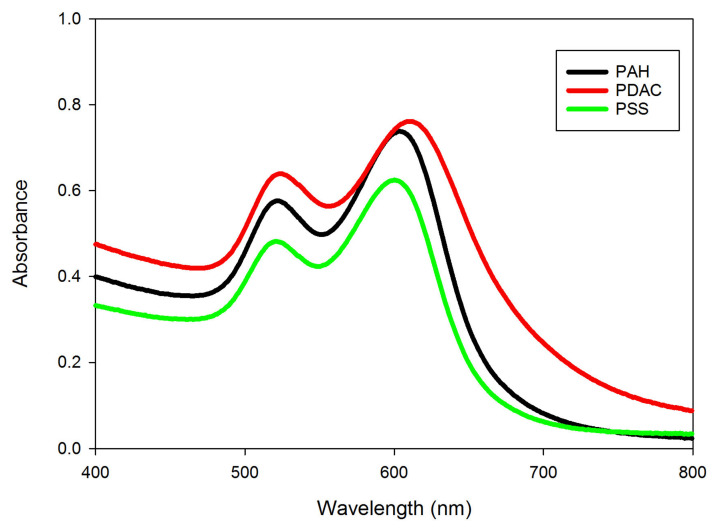
Absorption spectra of GNRs modified with PSS, PAH, and PDAC after functionalization.

GNRs, ~25 nm in width and ~47 nm in length, from Nanopartz were modified with PSS, PDAC, or PAH. Following this, the characterization of the GNRs was carried out using a UV-Vis spectrophotometer. GNRs modified with anionic PSS, cationic PDAC, and cationic PAH (called PSS-GNRs, PDAC-GNRs, and PAH-GNRs, respectively) had a transverse surface plasmon resonance (SPR_T_) peak at ~520 ± 3 nm. We found that the longitudinal surface plasmon resonance (SPR_L_) peaks of PSS-GNRs and PAH-GNRs were similar (at ~600 and ~604 nm, respectively). However, the SPR_L_ of the PDAC-GNRs was located at ~612 nm (Figure 2). The concentrations of all types of GNRs used in this work were adjusted to have a value of 1.0 at OD_605nm_ (the wavelength of the original maximum light absorption of GNRs before coating with polymers). In this case, at a laser wavelength of 635 nm, the absorbance was 0.53 for PSS-GNRs, 0.62 for PAH-GNRs, and 0.85 for PDAC-GNRs after their functionalization with the polyelectrolytes and the adjustment of their concentrations. Therefore, the PDAC-GNRs would be expected to absorb about 1.6× as well as the PSS-GNRs. The optical properties of PSS-GNRs and PDAC-GNRs can be clearly differentiated from one another, but those of PAH-GNRs are of an intermediate nature.

It is interesting to note that the first layer on the surface of all the GNRs was PSS, followed by the second layer of PDAC for PDAC-GNRs or PAH for PAH-GNRs. Therefore, both PDAC-GNRs and PAH-GNRs had a double layer of polyelectrolytes. In contrast, PSS-GNRs had only one layer (of PSS) on their surface. The zeta potentials measured from the DLS of the prepared PSS-GNRs, PDAC-GNRs, and PAH-GNRs were −40, +28, and +37 mV, respectively. These values confirm that the GNRs were successfully coated with PSS, PDAC, and PAH.

### 3.2. Effect of Different Surface Coatings of GNRs on Photothermal Conversion Efficiency

**Figure 3:**
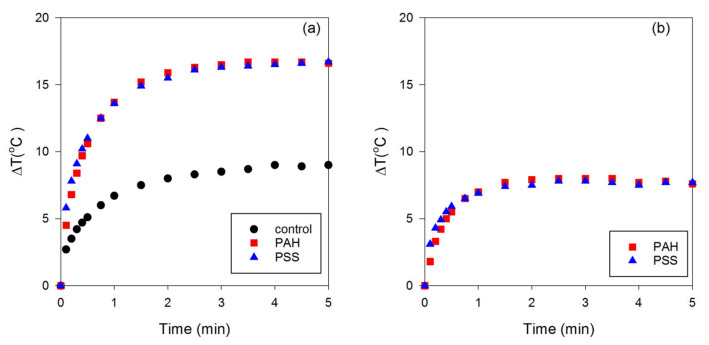
Increase in temperature of Milli-Q water (control), PSS-GNRs, and PAH-GNRs (30 µL) after exposure to a 100 mW laser (at a wavelength of 635 nm). The changes in temperature were collected after 0 to 5 min of laser exposure of the sample solution. (**a**) Total temperature increase in the system and (**b**) temperature increase after subtracting baseline due to the well plate and presence of the thermocouple.

Next, the photothermal conversion efficiencies of the PSS-GNRs and PAH-GNRs were studied by irradiating them with a laser. It is important to note here that it is the photothermal conversion efficiency of surface-modified GNRs, not that of the underlying GNRs themselves, that is the vital parameter for therapeutic treatment. As previously mentioned in the Introduction, the original GNRs have a toxic cationic detergent, CTAB, on their surface. Therefore, we did not investigate the photothermal conversion efficiencies of such GNRs. A small volume (30 µL in a 96-well plate) of PSS-GNRs and PAH-GNRs was used in the first experiment. Milli-Q water irradiated under the same conditions was used as the control sample. The temperature of the GNRs irradiated with a 635 nm laser at a laser power of 100 mW was recorded using a digital thermocouple for 5 min. The data are plotted in Figure 3. It is clear that the presence of the nanorods has raised the efficiency of heating; however, PSS-GNRs and PAH-GNRs have equal effects, notwithstanding their slightly different absorptances (0.53 and 0.62) at 635 nm. Figure 3 demonstrates that the temperature initially increases rapidly and then plateaus after ~2 min. After that time, convective heat loss to the environment is approximately equal to the energy deposited by the laser.

The starting temperature of the samples was ~23–25 °C. Maximum temperatures of ~40.6 °C (photothermal conversion efficiency = 2.50%) and 42.2 °C (photothermal conversion efficiency = 2.75%) were detected in PSS-GNR and PAH-GNR suspensions, respectively, after laser exposure. This provides a ΔT of the order of 18 °C. Note that room temperature was used here as the starting point for convenience. The application of the laser to samples held at body temperature (36 °C) would have produced a slightly smaller ΔT due to enhanced convective heat transfer to the environment. In addition to the change in temperature after irradiation, the photothermal conversion efficiency of PSS-GNRs and PAH-GNRs was similar; this could result from their similar SPR_L_, as discussed previously. The increase in the temperature of water was much lower than that of PSS-GNRs and PAH-GNRs. The maximum temperature detected in water was ~32.9 °C (photothermal conversion efficiency ~1.3%). It is important to emphasize that water has a very low light absorption at 653 nm, and therefore, the increase in temperature is likely due to some absorption by the sides of the polymer well rather than absorption by the water itself. However, a small volume (30 µL) was used in this experiment. This might have influenced the rise in water temperature after irradiation. Furthermore, the small volume used in this investigation might impact the increments ΔT of PSS-GNRs and PAH-GNRs, resulting in no temperature difference in either sample after irradiation.

**Figure 4:**
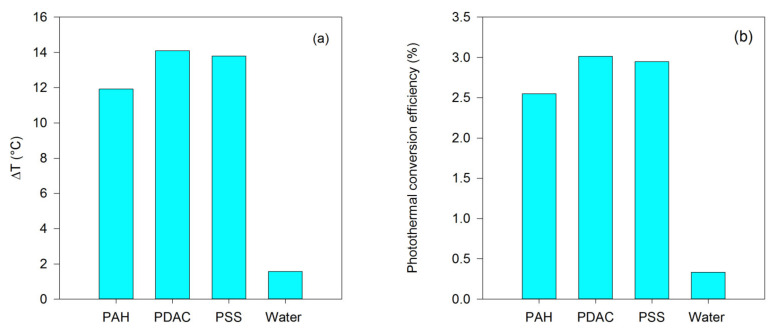
(**a**) Temperature difference and (**b**) photothermal conversion efficiency of PAH-GNRs, PDAC-GNRs, PSS-GNRs, and water (100 µL) after irradiation with 650 mW of a 653 nm laser for 5 min. The irradiation of water was performed as a control experiment.

To investigate the influence of the volume on the change in temperature, we increased the volume of the liquid to 100 µL but halved the concentration of the nanorods. The increases in temperature were low, so we also increased the laser power to 650 mW. The results (Figure 4) indicate that the highest temperature after 5 min was obtained for the PDAC-GNRs. This would be expected from the significantly higher absorbance at the laser wavelength compared to the other two types of GNR. However, the ranking of the photothermal conversion efficiencies of the PSS-GNRs and PAH-GNRs is reversed relative to their 635 nm absorbances in Figure 2. As shown in Figure 4, the temperature increase for PAH-GNRs (two layers) was lower than that for PSS-GNRs (one layer), even though both PAH-GNRs and PSS-GNRs exhibit maximum light absorption at about the same wavelength (at 600 nm) and the absorptance of PSS-GNRs at the laser wavelength is less than that for PAH-GNRs. This implies that the coating layer could impact the photothermal conversion efficiency of GNRs. It is worth noting that the use of these changed irradiation conditions (increased volume and laser power) allowed for the differentiation of the PSS- and PAH-GNRs. As expected, in the case of pure water, the increase in temperature was small after exposure to the laser. In addition, when the volume of water was increased from 30 to 100 µL, the temperature change was low, even when a higher output power (650 mW) was applied. When GNRs are present, however, the volume is also an important factor in heat generation (Figure 4). The shape of the aliquot is also important since, as its volume is increased, the contact area of the well sidewalls increases faster than the area of the air interface at the top, but there is a simultaneous decrease in the overall surface-to-volume ratio. Since conductive heat transfer takes place through the well material and convective heat transfer from the air interface, these factors will also affect the overall heat transfer.

The mismatch in wavelength between SPR_L_ and that of the laser used is another factor that will influence the amount of heating. If the plasmon resonance peak of GNRs is too far removed from the wavelength of the applied laser, the light absorption at the laser wavelength is low, resulting in a reduction in the final temperature. Therefore, the highest photothermal conversion efficiency, detected in PDAC-GNRs, could be because the SPR_L_ of PDAC-GNRs (~612 nm) is closer to the laser wavelength (635 nm) than those of PSS-GNRs and PAH-GNRs (~600 nm). A similar trend was reported in [19].

### 3.3. Numerical Simulations

**Figure 5:**
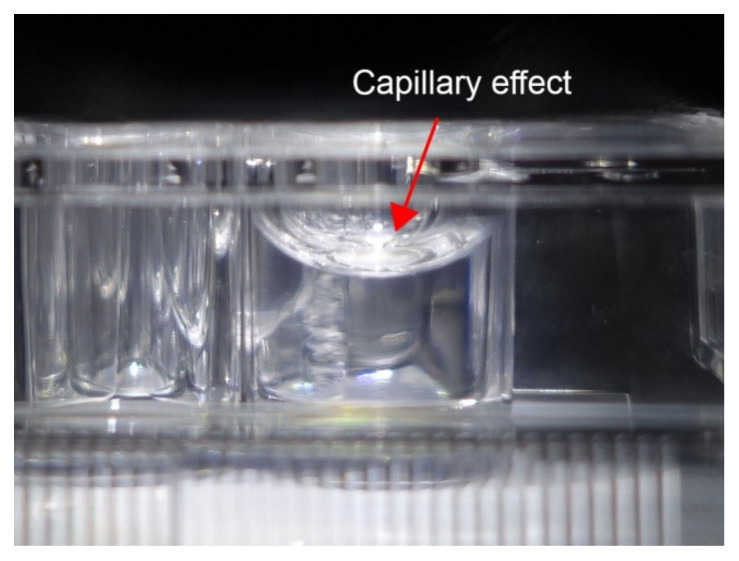
Capillary effect of the solution after the solution was pipetted into the well of a 96-well plate.

The numerical model for a 100 µL volume of a sample contained in a 96-well plate was run for various conditions. As for the physical experiment described earlier, the well diameter was 6.5 mm, and the average solution height corresponding to its volume in the dish container was 2.8 mm. Owing to the small diameter of the 96-well plate and the high surface tension of the sample solution, a capillary effect was observed, as seen in Figure 5. The adhesion force between the wall of the 96-well plate and the solution resulted in an increase in the solution level close to the wall of the well, which was higher than the solution level at the center. These factors were put into the numerical model.

**Figure 6:**
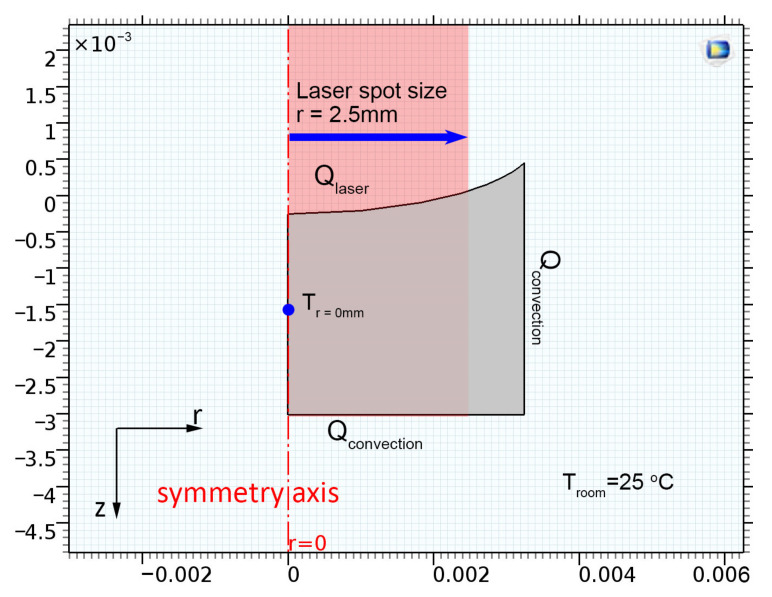
Boundary conditions of the axisymmetric model, where *x*- and *y*-axes are the length scales in meters.

Hence, the well has a cylindrical symmetry, and the simulation model was simplified and set as a two-dimensional axisymmetric model, as shown in Figure 6.

A laser with a spot size of 5 mm and a power intensity *I*_0_ of 650 mW (equivalent to an optical power of 33.1 kW/m^2^) was used to heat the solution. The beam was assumed to have a uniform radial distribution of energy and a sharp cutoff at its perimeter. The thermal energy imparted to the suspension by the laser irradiation, *Q*, was calculated using the Beer–Lambert law (Equation (2)) [22],

(2) $$Q\;=aI_{0}e^{-\alpha z}$$

where α  is the absorption coefficient (m^−1^), which was varied to match the results of the experiments, *z* is the length of the path through the suspension, $I_{0}\;$ is the laser power intensity (W m^−2^) at the surface of the solution, and the empirical factor ‘a’ has the magnitude and dimensions suitable to convert the intensity into units of power. The room temperature was maintained at ~25 °C. Heat losses due to the convective heat transfer around the well (${\;Q}_{\mathrm{losses}})$ were also included in the simulation. The transient heat transfer equation of the system [22] is shown in Equation (3).

(3) $$\rho C_{p}\frac{\partial T}{\partial t}+\nabla \left(-k\nabla T\right)=-\rho C_{p}u\cdot \nabla T+Q-{\;Q}_{\mathrm{losses}}$$

where *k* is the thermal conductivity (W m^−1^ K^−1^), *ρ* is the density (kg m^−3^), and C_p_ (J kg^−1^ K^−1^) is the heat capacity of Milli-Q water. Navier–Stokes equations were used to describe the velocity and temperature of the fluid. The boundary conditions at the bottom and vertical walls of the well were set to natural convective heat transfer with a constant room temperature of 25 °C. The top of the solution was exposed to air, causing heat loss to the surroundings due to convective heat transfer. The center of the well was exposed to the laser with a laser spot size diameter of 5 mm to match the experimental setup.

**Figure 7:**
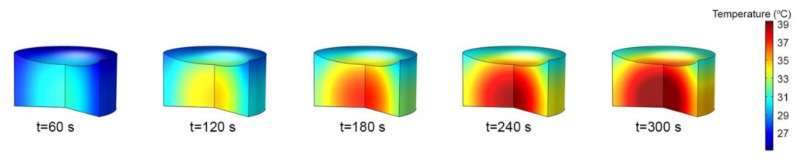
Time evolution of temperature in the solution (100 µL) of α  = 16 m^−1^.

As seen in Figure 7, heat was only absorbed by the GNR solution within the laser spot area and accumulated at the center of the well. Therefore, the hottest area could be observed in the middle of the solution. The temperature steadily decreased toward the wall because of the lower temperature at the wall of the well plate. Since the domain was small, heat transfer in the solution was dominated by conduction, and heat diffused radially outward toward the cooler walls of the cell. The meniscus of the liquid around the walls of the well exhibited the lowest temperature because it was located outside the laser spot area and was exposed to a significant heat sink (the well walls).

**Figure 8:**
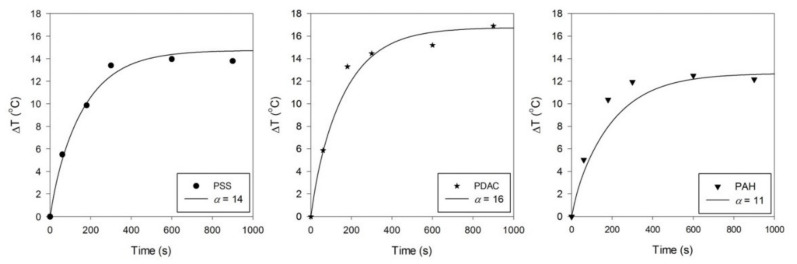
Temperature increase of 100 µL Milli-Q water with PSS-GNRs, PDAC-GNRs, and PAH-GNRs after exposure to a 650 mW laser (at a wavelength of 635 nm), compared with the simulation results (solid lines) measured at r = 0.001 m and z = −0.0015 m. The circle, star, and inverted triangle symbols present experimental data.

Comparisons between the experiment and simulation are shown in Figure 8 for the three different types of GNRs. As seen in Figure 8, after the GNR solutions (100 µL) were irradiated with a 650 mW laser, the temperatures increased sharply in the first 3 min, similar to those in the first experiment with the small-volume solution (30 µL; Figure 3). The 100 μL aliquots approached a quasi-steady state at around 5 min (Figure 8). The average temperature differences (ΔT) of the PDAC-GNR, PSS-GNR, and PAH-GNR solutions at 10 min were ~15.2, 14.0, and 12.5 °C, respectively. The simulation results show that the trend of increasing temperature in GNR solutions agrees well with the experimental results. The absorption coefficients α of the PSS-GNR, PDAC-GNR, and PAH-GNR solutions were iterated to match the experiment results and were found to be around 14, 16, and 11, respectively; however, this is not a perfect match for the ranking of the measured absorptances at 635 nm, which were 0.53, 0.85, and 0.62, respectively. Presumably, some scatter in the experimental results is responsible for obscuring the difference between the PSS-GNRs and the PAH-GNRs. The two coating layers of the PAH-GNRs might also have impacted the absorption coefficients. It might be worthwhile to further investigate this experimentally in the future.

As discussed previously, the coating layer could affect the photothermal conversion efficiency of GNRs, as seen in the case of PSS-GNRs and PAH-GNRs, which had the same SPRL. The simulation results also show that the value of the absorption coefficient of PAH-GNRs was lower than that of PSS-GNRs. Furthermore, the simulation results reproduce the change in the GNR temperature. The heat transfer was mainly due to conduction, both within the fluid and outward to the well plates. The hottest area of the solution was in the laser spot area. The lowest temperature was detected at the top capillary area of the solution because it was outside the laser spot area. The boundary conditions in a very small domain strongly affect the temperature of the GNR solutions; in particular, the high surface-to-volume ratio of the droplets tested and simulated in the present work causes the rate of heat loss to the environment to be very high. Consequently, an overall heating efficiency of only 3% was attained. This limits the increase in the temperature of the fluid to 15 °C at most when a 650 mW laser is used in this geometry. This would, however, still be sufficient to achieve a useful effect. The relation between the temperature difference and the absorption coefficient could be useful for predicting rising temperatures.

## 4. Conclusions

GNRs are potent optical absorbers, and their presence will lead to effective heating in fluids that are laser-irradiated at an appropriate wavelength. The surface coating of GNRs could impact the position of the longitudinal surface plasmon resonance and hence the photothermal conversion efficiency and needs to be considered. In addition, the geometry of the configuration being irradiated is very important. When, for example, very small volumes are being irradiated in a clear plastic well plate, at least 97% of the laser energy is lost as a result of conduction or convection to the environment or transmission through the sample, and only 3% is retained in the fluid aliquot. Furthermore, convective and conductive heat transfer will generate a temperature distribution of the test solution in the well [23]. Nevertheless, the temperature increase needed to induce hyperthermia can be readily reached after 5 min even in such geometries using irradiation with a CW laser of the appropriate wavelength and sufficient power. These insights and measurements may be useful when designing future studies of GNR-induced plasmonic heating in tissues or cell cultures.

## Data Availability

Not applicable.
